# Estimating Congenital Cardiac Surgical Need in Africa Using Geographic Distribution of Surgeons

**DOI:** 10.5334/aogh.4692

**Published:** 2025-06-25

**Authors:** Jordan Leith, Lamia Harik, Kevin R. An, Taylor Brashear, Robert N. Peck, Castigliano M. Bhamidipati

**Affiliations:** 1 Department of Cardiothoracic Surgery, Weill Cornell Medicine, New York, New York; 2 Center for Global Health, Weill Cornell Medical College, New York, New York; 3 Department of Medicine, Bugando Medical Centre, Mwanza, Tanzania; 4 Division of Cardiothoracic Surgery, Department of Surgery, Oregon Health & Science University, Portland, Oregon

**Keywords:** congenital heart disease, access to care, global health

## Abstract

*Background:* Access to congenital cardiac surgical care in Africa is limited and poorly characterized, with current assessments examining only the number of surgeons in individual countries compared to their respective national population.

*Objective:* To characterize geographic catchment areas in Africa served by the nearest congenital cardiac surgeon(s), estimate patient travel distance, and map both the incidence and unmet surgical need due to congenital heart disease (CHD).

*Methods:* Subnational population, CHD incidence, surgeon, and geographic data were collected from credible, publicly accessible sources. Quantum Geographic Information System was used to create a subnational map of Africa and conduct nearest neighbor analyses to determine the location and distance of each subnational region’s nearest cardiac surgeon. Catchment areas were defined and characterized. Incident CHD cases and surgical needs due to CHD were calculated and mapped at the subnational level across Africa.

*Findings:* There were 779 subnational regions from 54 countries included in this analysis. Africa was estimated to have 290 congenital cardiac surgeons located in 63 subnational regions corresponding to 63 catchment areas and 1,097,388 incident CHD cases annually. The average travel distance to a congenital cardiac surgeon was 324.40 km (201.57 miles). The ratio of incident CHD to total surgical case capacity was 20.79. Congenital cardiac surgical need was not limited to areas of high incidence and was compounded by distance to the nearest surgeon, with the greatest need occurring in the Congo Basin and Horn of Africa.

*Conclusions:* Access to congenital cardiac surgery is limited in Africa with the capacity to surgically treat less than 5% of annual CHD cases. Surgical need is exacerbated by the geographic distribution of surgeons, which requires patients to travel great distances.

## Introduction

Current estimates report that congenital heart disease (CHD) occurs in one out of every 100 live births globally [1–3], with 70% requiring medical and/or surgical intervention within the first year of life [4]. However, in low‑ or middle‑ income countries (LMICs), approximately 90% of patients are denied or do not receive standard of care [3], with one in three infants dying in the first month of life [5] due to limited access to centers with cardiac surgical capabilities [6].

Compared to high‑income countries such as the United States, access to cardiac surgical care in LMICs is limited—with countries in Sub‑Saharan Africa having both the fewest surgeons and centers [6, 7]. Contemporary studies assessing global access to congenital cardiac surgical care have presented the need gap by evaluating the number of surgeons or centers, on the basis of population [6, 7]. Studies of access to congenital cardiac surgical care in Africa have been limited to these generic global assessments. There has been no work that maps the incidence of CHD, characterizes geographic catchment areas, and considers travel distance for congenital cardiac surgical care as has been done in the US [8, 9]. This is especially pertinent in Africa given its vast geography as well as topography and potential to optimally locate centers with congenital cardiac surgical capabilities (the US is ~3.8 million square miles and Africa is ~11.7 million square miles).

The aim of this study is to utilize publicly available geographic data, estimates of population, disease incidence, and surgeon demographics in Africa to characterize geographic catchment areas serviced by the nearest congenital cardiac surgeon(s), estimate travel distance, and co‑register the incidence of CHD to truly establish the unmet surgical need.

## Material and Methods

This cross‑sectional study did not involve human subjects and was conducted using publicly available data sources; therefore, IRB approval and informed consent were not required.

### Definitions

Subnational region: The largest defined geographic designation in a country below the national level (analogous to a state or province).

Catchment area: The geographic region a congenital heart surgeon may receive a referral from, defined in this study as the subnational regions to which a congenital cardiac surgeon is closest, regardless of national borders.

Choropleth map: A map displaying the distribution of CHD incidence or CHD surgical need.

Incident case to capacity ratio (ICCR): An annualized, calculated ratio of CHD incidence to congenital cardiac surgical case capacity. Ratios greater than one suggest that a region does not have the capacity to meet the surgical demand.

CHD surgical need: The distance of a subnational region from the nearest congenital cardiac surgeon, calculated by the geospatial nearest neighbor analysis, multiplied by the ICCR of the respective catchment area. This computation is a surrogate that represents the challenges patients face, both traveling from distant regions and obtaining definitive screening/referral for CHD.

### Data sources

Using the Institute for Health Metrics and Evaluation (IHME) global burden of disease (GBD) 2019 tool, CHD incidence was collected from the Global Health Data Exchange [10]. The full IHME methodology of defining CHD and calculating disease incidence has been previously described [11, 12]. Briefly, CHD was defined as a composite of five pathologic subcategories based on anatomical characteristics and the treatment requirements of each condition: (1) single ventricle and single ventricle pathway defects, (2) complex congenital heart defects excluding single ventricle and single ventricle pathway defects, (3) malformations of great vessels, congenital valvular heart disease, and patent ductus arteriosus, (4) ventricular septal defect and atrial septal defect, and (5) other congenital cardiovascular anomalies [11].

As reported previously, the Cardiothoracic Surgery Network (CTSnet) was queried [7] to determine the number of congenital cardiac surgeons and their geographic region of practice for each country in Africa [13]. For greater fidelity, a personal communication with the president of the Pan African Society of Cardiothoracic Surgery yielded the most accurate list of known centers in Africa with cardiac surgery capabilities. This list was cross‑referenced with CTSnet data.

Countries were divided into subnational regions. Shapefiles for these regions were collected by country from the Global Administrative Areas (GADM) [14] Database and merged to form a subnational map of Africa in Quantum Geographic Information System (QGIS) 3.2 Lima.

Adult and pediatric (under the age of 20) populations for these subnational regions were gathered using each country’s census data. If census data for a country were not available or the country did not break down its population by region, population data were collected from the Humanitarian Data Exchange [15], an open‑source database maintained by the United Nations Office for the Coordination of Human Affairs (exceptions: subnational populations were unavailable for Equatorial Guinea and Republic of the Congo, therefore nationwide metrics were used) [16].

### Outcomes of interest

Outcomes were stratified into three geographic levels: continental (composed of all subnational regions), catchment area, and subnational. At the continental level, outcomes included population, number of congenital cardiac surgeons with the corresponding number of catchment areas, CHD incidence, the average distance to a congenital cardiac surgeon, and ICCR. At the level of catchment areas, outcomes included the population served, the average distance to congenital cardiac surgeon(s), and the ICCR. At the subnational level, outcomes included the distribution of CHD cases and their surgical need.

### Analysis

Population, incidence of CHD, and congenital cardiac surgeons were merged into QGIS according to their respective subnational region. Subnational CHD incident cases were calculated for congenital heart disease (Equation 1). A choropleth map was then created depicting the incidence of CHD for each subnational region across Africa, stratified by decile.


$${\text{Incident Cases}}_{\text{CHD}}=\text{Subnational Population}\times \frac{{\text{Incidence}}_{\text{CHD}}}{100,000}$$ 1

A nearest neighbor analysis was conducted to determine the nearest congenital cardiac surgeon(s) and corresponding distance for each subnational region in order to construct catchment areas as in prior US‑based studies [8]. The population and the average distance a patient would need to travel were calculated for each catchment area as described previously [8, 9].

ICCRs were calculated for each catchment area (Equation 2). The annual average case volumes of congenital cardiac surgeons were derived from Society for Thoracic Surgeons or the American Association for Thoracic Surgery (STS/AATS) task force surveys and extrapolated for use in Africa [17].


$$\begin{array}{l} \text{Incident Case to Capacity}\;{\text{Ratio}\;}_{\text{Catchment Area}\;}\\ \;=\frac{\sum_{n=i}^{j}\text{Subnational}\;{\text{Population}}_{i}\times \frac{{\text{Incidence}}_{CHD_{i}}}{100,000}}{\;\#\text{Congenital}\;\text{Cardiac}\;\text{Surgeons}\;(\text{Catchment}\;\text{Area})\times \text{Avg}.\text{Annual}\;\text{Case}\;\text{Vol}.} \end{array}$$ 2

CHD surgical need for each subnational region was calculated (Equation 3). A second choropleth map was generated displaying subnational CHD surgical need, stratified by decile, with lines linking each subnational region to the subnational region of the nearest congenital cardiac surgeon. Subnational regions linked to the same surgeon(s) were within the same catchment area.


$$\begin{array}{l} \;\text{Surgical}\;{\text{Need}}_{\text{CHD}-\text{Dist}}\\ \;=\frac{\left(\sum_{n=i}^{j}\text{Subnational}\;{\text{Population}}_{i}\times \frac{{\text{Incidence}}_{{CHD}_{i}}}{100,000}\right)\times \frac{\text{Distance}\;\text{to}\;\text{Catchment}\;\text{Area}\;\text{Surgeon}\;(\text{km})}{100}}{\;\#\text{Congenital}\;\text{Cardiac}\;\text{Surgeons}\;(\text{Catchment}\;\text{Area})\times \text{Avg}.\text{Annual}\;\text{Case}\;\text{Vol}.} \end{array}$$ 3

## Results

### Continent

The African continent was estimated to have a population of 1,298,333,309 people, 658,283,537 adults (50.7%) and 640,049,772 (49.3%) children under the age of 20. There were 290 congenital cardiac surgeons practicing in 63 subnational regions (corresponding to 63 catchment areas). There were 1,097,388 incident CHD cases annually. The average distance to the nearest congenital cardiac surgeon, weighted by population, was 324.40 km (201.57 miles). The ICCR for the African continent was 20.79. Taking the reciprocal of the continental ICCR and generating a ratio of case capacity to incident cases revealed that only 4.81% of CHD cases could be accommodated by current surgeons.

### Catchment areas

**Figure 1:**
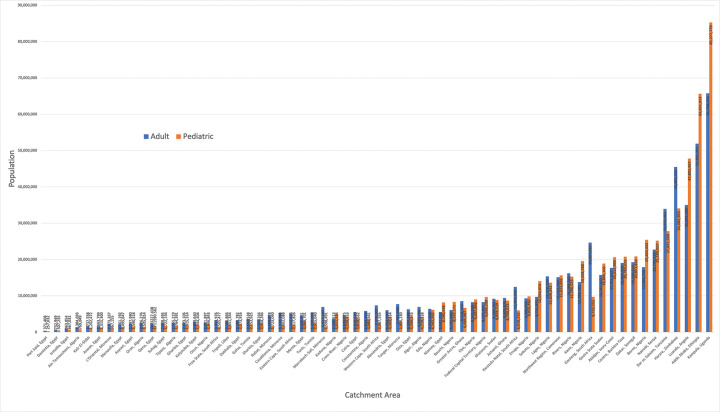
Populations served by the 63 congenital cardiac surgical catchment areas across Africa.

The population of each catchment area ranged from 628,440 individuals (served by one congenital cardiac surgeon in Port Said, Egypt) to 151,025,883 individuals (served by one congenital cardiac surgeon in Kampala, Uganda). The populations of each catchment area stratified by age group are shown in Figure 1.

**Figure 2:**
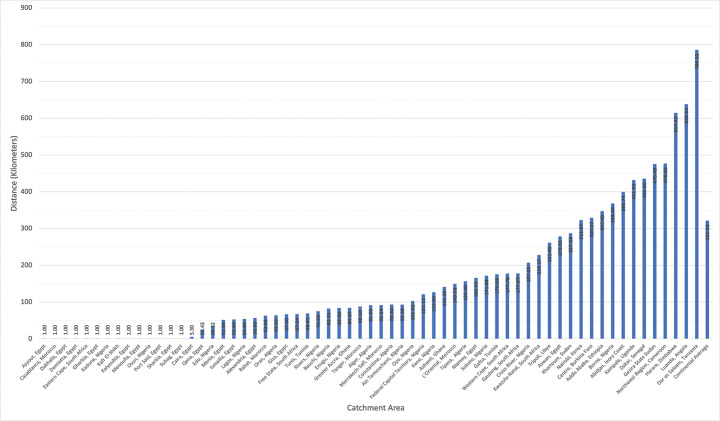
Average distance a patient in each catchment area would travel to see a congenital cardiac surgeon, weighted by population.

The average distance a patient would need to travel (Figure 2) to see the congenital cardiac surgeon(s) in their catchment area, weighted by population, ranged from 1 km (0.62 miles) to 786.23 km (488.54 miles).

**Figure 3:**
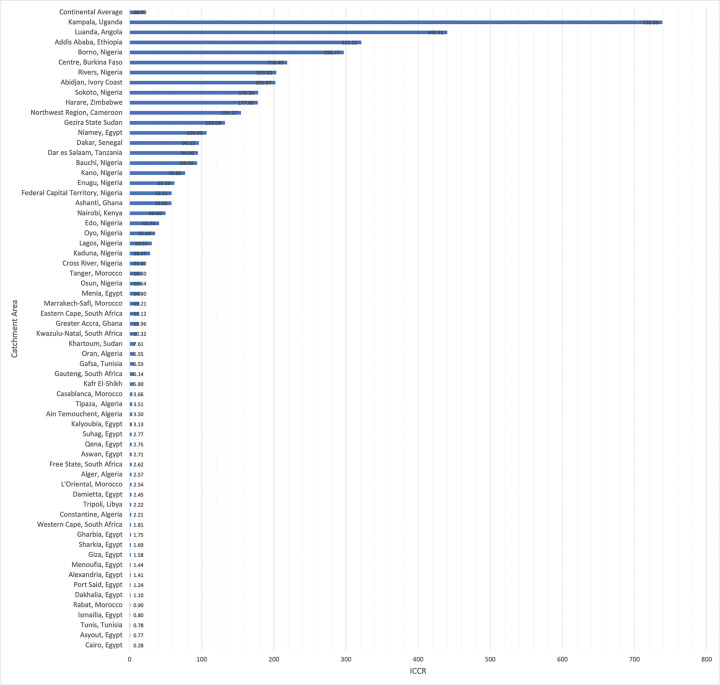
Incident case to capacity ratio for each of the 63 congenital cardiac surgical catchment areas across Africa. *ICCR: incident case to capacity ratio.*

The ICCR for each catchment area ranged from 0.28 for regions served by Cairo, Egypt to 738.39 for regions served by Kampala, Uganda (Figure 3). Additionally, the ICCR for each catchment area was also generated using the alternative assumption that local surgeons might only be able to meet 75% or 50% of average US case volumes. These results are depicted in Supplementary Figure 1 and Supplementary Figure 2, respectively.

### Subnational regions

**Figure 4:**
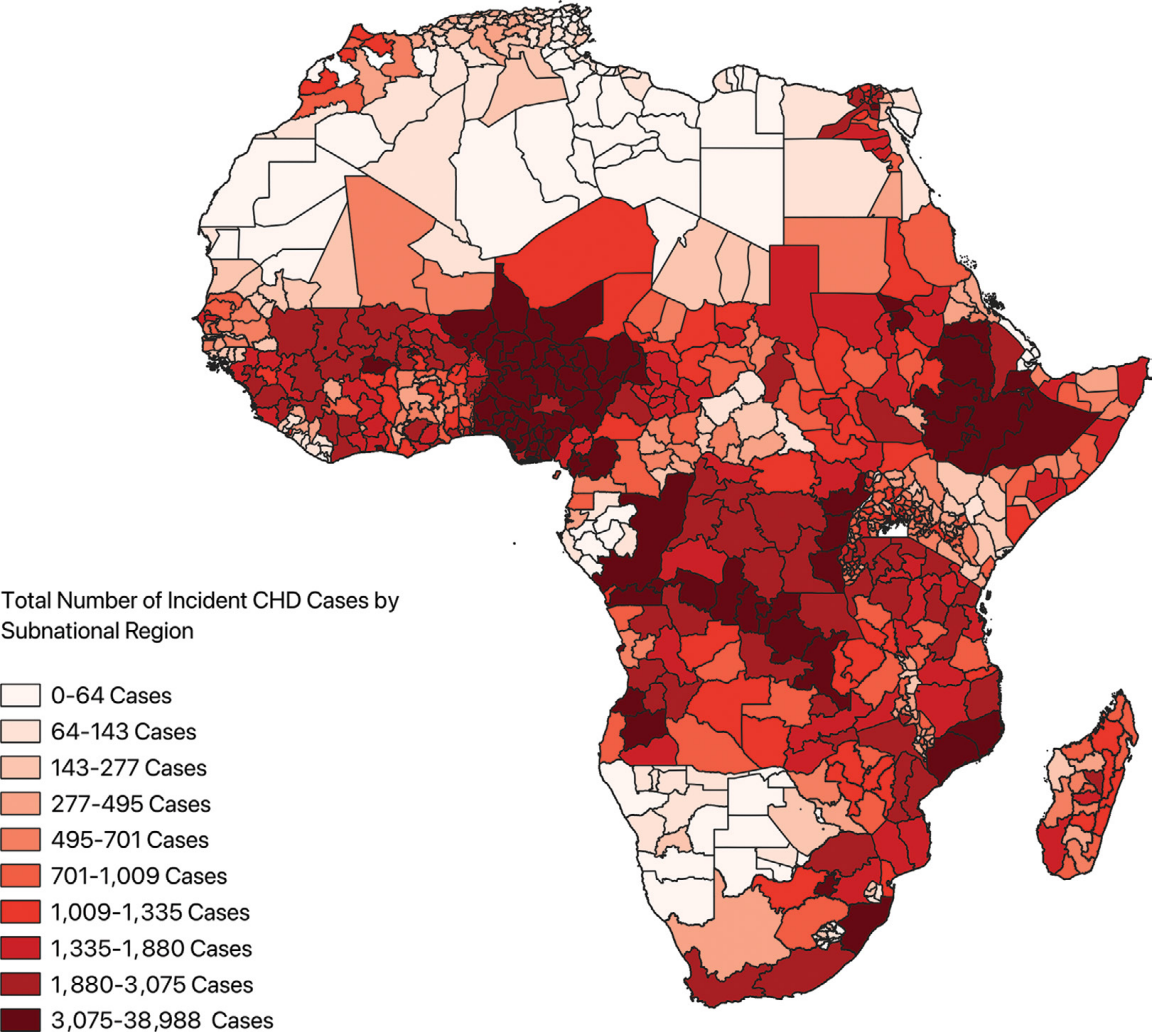
Choropleth map depicting incident cases of congenital heart disease across the subnational regions of Africa. *CHD: congenital heart disease.*

A total of 779 subnational regions from 54 countries were included in the analysis. The estimated median was 700.81 (IQR: 211.14–1616.77) and the mean was 1406.89±89.73 incident cases of CHD across all subnational regions. The estimated annual number of index CHD cases by subnational region ranged from 1.19 cases (min‑max: 0.87–1.63) (Sowa Town, Botswana) to 38,987.86 cases (min‑max: 29,378.62–51,604.00) (Oromia, Ethiopia). A choropleth map depicting the incidence of CHD in each subnational region demonstrated the highest case density occurring in West Africa, the Horn of Africa, and along the coast of Southeast Africa (Figure 4). Additionally, rates of the incidence of CHD for each catchment area were tabulated (Supplementary Figure 3).

**Figure 5:**
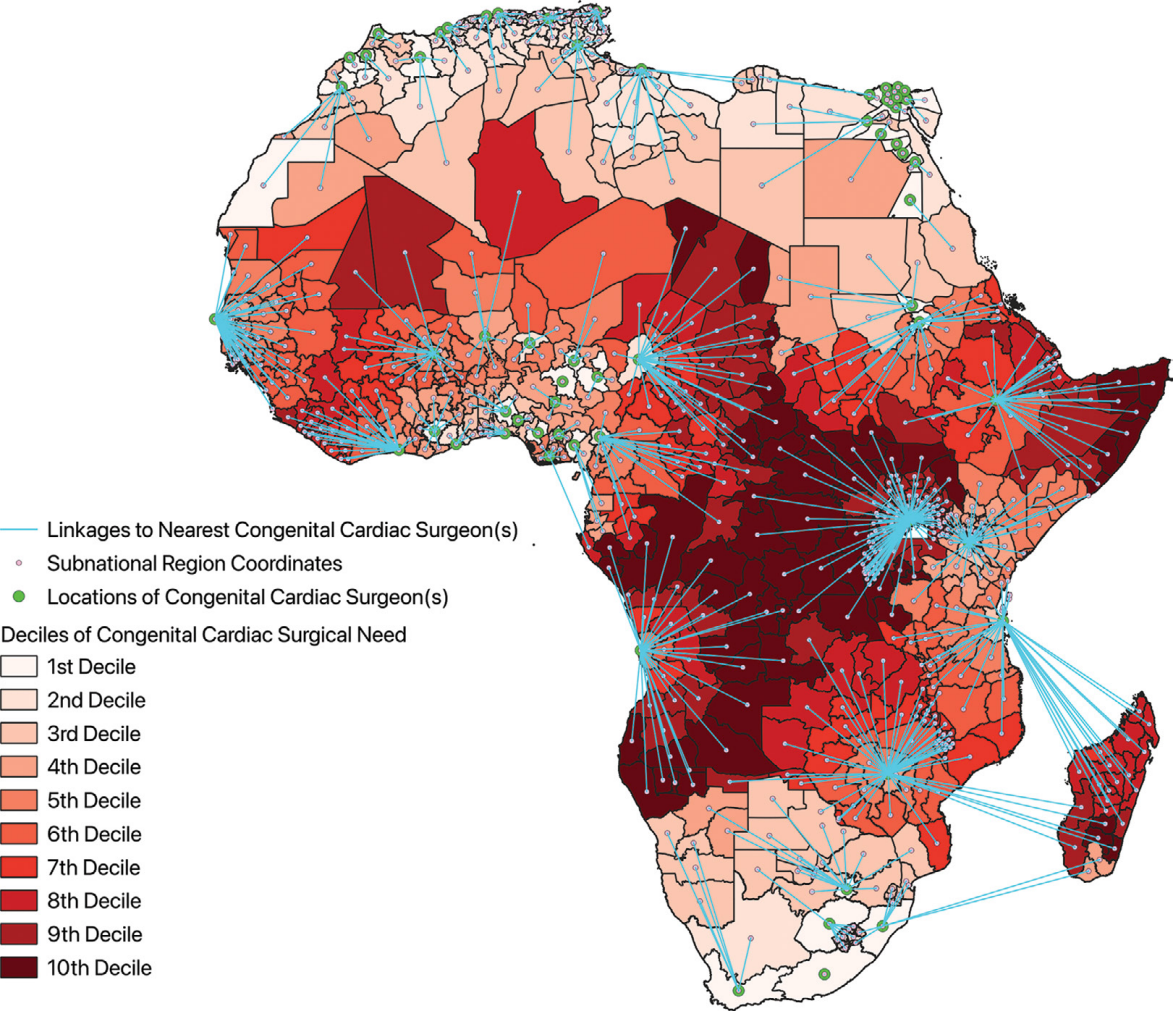
Choropleth map of congenital cardiac surgical need across the subnational regions of Africa with linkages to the nearest congenital cardiac surgeon.

Congenital cardiac surgical need for each subnational region, incorporating distance from the nearest congenital cardiac surgeon, is shown in Figure 5, where blue lines link each subnational region to the location of the nearest congenital cardiac surgeon. Areas of greatest CHD surgical need occurred in the Horn of Africa along the coast of the Indian Ocean and centrally through the Congo Basin.

## Comment

Prior work in estimating the clinical need gap in Africa has focused on traditional epidemiologic metrics to assess the limited access to congenital cardiac surgical care. We are the first to demonstrate that the problem is more complex by estimating that less than 5% of incident CHD cases can be accommodated by current surgical capacity and highlighting the role geography plays in limiting access to congenital cardiac surgery. In our work, examining 779 subnational regions comprising 63 catchment areas across Africa, we found that the average distance to travel for care by a congenital cardiac surgeon was 324.40 km (201.57 miles). Further, we demonstrate that the highest CHD surgical need is not isolated nor collocated to areas with the highest incidence of CHD but occurs in subnational regions that are most distant from the nearest congenital cardiac surgeons. These results suggest that increasing the number of congenital cardiac surgeons within Africa is imperative, but their geographic orientation is perhaps just as important.

Previous estimates of national cardiac surgical capacity in Africa have restricted their focus to standardizing cardiac centers (median: 0 centers per million people, IQR: 0–5) [6] and cardiac surgeons (mean: 0.19 surgeons per million people) [7] by population. Transportation alone becomes prohibitive for patients given the cost [18, 19] and lack of paved roads [20, 21]. Moreover, studies of the geographic distribution of congenital cardiac surgeons have been limited to high‑income countries such as the US [8, 22]; an analysis of current patient travel patterns in the US found that the median distance traveled to see a congenital cardiac surgeon is 38.5 miles [9]. A geographical analysis of congenital heart surgery patients based on population distribution, case complexity, and comprehensive care provision in the US has led to a proposal of regionalization of congenital cardiac surgery with fewer and more optimally relocated centers [8]. Similarly, since congenital cardiac surgery is needed direly and surgeons are distributed disparately, our work suggests that Africa also requires strategic structuring and geographic translocation of resources to areas of greatest need—easier said than done.

The dearth of funding that cardiac surgery receives from the largest donor of global health initiatives in the world (the US government) is noteworthy [23]. In 2016, 0.26% of the federal US budget (~$10.3 billion) was earmarked for global health initiatives [24]. Of these, funds directed to noncommunicable diseases, including cardiovascular disease and surgical conditions, jointly received only 3% of the earmarked allocations [25–27] (~$3 M). In contrast, HIV, tuberculosis, and malaria received over 50% of these funds [26]. In Africa, the mortality associated with cardiovascular and surgical conditions accounts for five times as many deaths as HIV, tuberculosis, and malaria combined [28–30]. While this is due to the groundbreaking advancements in the management of infectious diseases and efforts of global health champions over the past four decades, perhaps it is time to restructure funding models to better accommodate non‑infectious diseases.

In the absence of sizable global health funding as well as independent, sustainable, and sufficient congenital cardiac surgical services, there has been emphasis on utilizing non‑governmental organizations (NGOs) to mitigate the need gap for access to congenital cardiac surgeons in LMICs. Previous studies have identified approximately 81 NGOs providing congenital cardiac surgery in 96 LMICs [31, 32] performing an average of 8,047 operations annually across the globe, with the median performed by individual NGOs being 100 cases [32]. While this is important and improves the lives of recipients, from a system’s perspective, given our estimate of 1,045,000 annual incident cases of CHD unmet by local capacity, the need gap in Africa remains cavernous despite NGO involvement. Moreover, NGOs face unique challenges limiting their efficacy, including mistrust between transient NGO and local staff, post‑operative follow‑up, management of complications when surgical staff reside overseas, and disparate medical record keeping [33, 34].

It is clear from the efforts of these NGOs and the surgical mission work that is performed by smaller groups of surgeons that establishing equitable congenital cardiac surgical care across Africa is a long‑term goal. However, current efforts are often uncoordinated and only transiently address resources, staffing, and setting. These are three mission critical elements in developing a functional, long‑term surgical system that can meet the needs of patients with CHD. One of the most promising methods of developing congenital cardiac surgical workforces has been the establishment of *twinning programs* in which cardiac programs of excellence in resource‑rich settings are paired with developing cardiac programs in resource‑limited settings [35, 36]. This longitudinal partnership (typically >5 years) fosters bidirectional learning and skill development, which can include formal instruction (i.e. fellowship, perfusionist training, etc.), material support aimed at sustainability, and preparing developing centers to become training centers themselves [36], while minimizing challenges of the NGO model.

While these twinning models are promising, they are often limited by the constraints of a single institution and its cardiac surgical faculty, which also have competing interests to practice domestically to keep their departments afloat. For this reason, it might be beneficial to concert the efforts of NGOs, academic institutions, and individual surgeons through a professional organization such as the STS or the AATS to invest in a smaller subset of partners year‑round until they become training centers. This would effectively allow surgeons and local partners to pool expertise, staff, and resources in a singular space until it is ready to operate independently. Once a center is established, this program could expand to subsequent partners in an iterative process. An important derivative benefit is that the American Board of Thoracic Surgery (ABTS), STS, and AATS could leverage these *twinning programs* as a training conduit while also helping African nations better serve their populations.

Providing congenital cardiac care to African patients in remote locations will require innovation while new hospitals with these capabilities are established. General and surgical subspecialties have overcome similar challenges with teams of local surgeons leading mobile surgical units [37, 38]. Adoption of these ideas in the congenital cardiac context could be used to augment ongoing screening missions to remote locations performed by NGOs [39] and provide routine follow‑up care. Ultimately, while intended as a temporizing measure, this would also provide graduates from nascent African cardiac programs a flexible practice in addition to expanding access to care [37]. However, if mobile surgical units are not feasible because congenital cardiac surgery is so resource and staffing intensive, focus could also be shifted towards developing patient transport networks to the nearest center with congenital cardiac surgical capabilities.

## Limitations

Our analysis has several limitations. We fully acknowledge that our methodology of collecting surgeon data from CTSnet is imperfect. However, this methodology has been used previously in other large‑scale geographic analyses of access to cardiac surgical services [7] and is necessary as no other continent‑wide registry of cardiothoracic surgeons exists. It also avoids the availability bias inherent in relying on information from regional contacts.

Catchment areas were constructed irrespective of national borders; assuming patients would be able to cross borders without difficulty due to medical need may not reflect geopolitical reality. Additionally, we calculated distances in a linear point‑to‑point fashion, not accounting for the actual road/transit networks patients might use, which are likely further and inefficient. However, as over 50% of roads are unpaved and the cost of air travel is prohibitive for a majority of citizens [21], this method is a reasonable approximation. Moreover, we assumed each surgeon could meet the average US case volume and that this case volume was composed solely of CHD. This is likely not the case, given the high incidence of rheumatic heart disease, and specialization in congenital caseloads is rare in LMICs. Finally, we assumed that the national estimates of CHD incidence were homogeneous throughout a country.

## Conclusions

Across Africa, access to congenital cardiac surgical care is limited. Attention should be paid to the geographic distribution of surgeons as limited access is compounded by geographic distance. Potential solutions provide an opportunity for innovation and teamwork by local and international surgeons.

## Figures and Tables

**Table:** 

AATS	American Association for Thoracic Surgery
ABTS	American Board of Thoracic Surgery
CHD	Congenital Heart Disease
CTSnet	The Cardiothoracic Surgery Network
GADM	Global Administrative Areas
GBD	Global Burden of Disease
ICCR	Incident Case to Capacity Ratio
IHME	Institute for Health Metrics and Evaluation
LMIC	Low‑ to Middle‑Income Country
NGO	Non‑governmental Organization
STS	Society of Thoracic Surgeons
QGIS	Quantum Geographic Information System

## Additional Files

The additional files for this article can be found as follows:

Supplementary Figure 1. ICCR for each of the 63 Congenital Cardiac Surgical Catchment Areas Across Africa Operating at 75% U.S. Case Capacity.

Supplementary Figure 2. ICCR for each of the 63 Congenital Cardiac Surgical Catchment Areas Across Africa Operating at 50% U.S. Case Capacity. *ICCR: Incident Case to Capacity Ratio*.

Supplementary Figure 3. Tabulated Rates of CHD Incidence for Each Congenital Cardiac Surgical Catchment Area, *CHD: Congenital Heart Disease*.

## References

[r1] Hoffman JIE, Kaplan S, Liberthson RR. Prevalence of congenital heart disease. Am Heart J. 2004;147(3):425–439. doi:10.1016/j.ahj.2003.05.003. 14999190

[r2] Kang L, Cao G, Jing W, Liu J, Liu M. Global, regional, and national incidence and mortality of congenital birth defects from 1990 to 2019. Eur J Pediatr. 2023;182(4):1781–1792. doi:10.1007/s00431-023-04865-w. 36781460

[r3] van der Linde D, Konings EEM, Slager MA, et al. Birth prevalence of congenital heart disease worldwide: A systematic review and meta‑analysis. J Am Coll Cardiol. 2011;58(21):2241–2247. doi:10.1016/j.jacc.2011.08.025. 22078432

[r4] Sandoval N, Kreutzer C, Jatene M, et al. Pediatric cardiovascular surgery in South America: Current status and regional differences. World J Pediatr Congenit Heart Surg. 2010;1(3):321–327. doi:10.1177/2150135110381391. 23804888

[r5] Hewitson J, Zilla PPT. Children’s heart disease in sub‑Saharan Africa: Challenging the burden of disease. SAHJ. 2017;7(1):18–29. doi:10.24170/7-1-1964.

[r6] Vervoort D, Babar MS, Sabatino ME, et al. Global access to cardiac surgery centers: Distribution, disparities, and targets. World J Surg. 2023;47(11):2909–2916. doi:10.1007/s00268-023-07130-1. 37537360

[r7] Vervoort D, Meuris B, Meyns B, Verbrugghe P. Global cardiac surgery: Access to cardiac surgical care around the world. J Thorac Cardiovasc Surg. 2020;159(3):987.e6–996.e6. doi:10.1016/j.jtcvs.2019.04.039. 31128897

[r8] Welke KF, Pasquali SK, Lin P, et al. Theoretical model for delivery of congenital heart surgery in the United States. Ann Thorac Surg. 2021;111(5):1628–1635. doi:10.1016/j.athoracsur.2020.06.057. 32860751

[r9] Welke KF, Pasquali SK, Lin P, et al. Hospital distribution and patient travel patterns for congenital cardiac surgery in the United States. Ann Thorac Surg. 2019;107(2):574–581. doi:10.1016/j.athoracsur.2018.07.047. 30248321

[r10] Global Health Data Exchange | GHDx. IHME | Global Health Data Exchange. Accessed November 15, 2023. https://ghdx.healthdata.org/.

[r11] Congenital birth defects | Institute for Health Metrics and Evaluation. Accessed April 25, 2024. https://www.healthdata.org/gbd/methods-appendices-2021/congenital-birth-defects-0.

[r12] Protocol for the Global Burden of Diseases, Injuries, and Risk Factors Study | IHME. Accessed April 25, 2024. https://www.healthdata.org/sites/default/files/files/Projects/GBD/March2020_GBD%20Protocol_v4.pdf.

[r13] Search for Surgeons | CTSNet. Accessed April 25, 2024. https://www.ctsnet.org/surgeons.

[r14] Database of Global Administrative Areas (GADM). Accessed November 15, 2023. https://gadm.org/.

[r15] The Humanitarian Data Exchange. Accessed November 13, 2023. https://data.humdata.org/.

[r16] The Centre for Humanitarian Data – Connecting people and data to improve lives. Accessed April 25, 2024. https://centre.humdata.org/.

[r17] Shemin RJ, Ikonomidis JS. Thoracic surgery Workforce: Report of STS/AATS thoracic surgery practice and access Task Force—Snapshot 2010. J Thorac Cardiovasc Surg. 2012;143(1):39–46.e6. doi:10.1016/j.jtcvs.2011.10.022. 22172751

[r18] Raj M, Paul M, Sudhakar A, et al. Micro‑economic impact of congenital heart surgery: Results of a prospective study from a limited‑resource setting. PLoS One. 2015;10(6):e0131348. doi:10.1371/journal.pone.0131348. 26110639 PMC4482148

[r19] Price J, Willcox M, Dlamini V, et al. Care‑seeking during fatal childhood illness in rural South Africa: A qualitative study. BMJ Open. 2021;11(4):e043652. doi:10.1136/bmjopen-2020-043652. PMC8094335 33926978

[r20] Graff T. Spatial Inefficiencies in Africa’s Trade Network. National Bureau of Economic Research; 2019:w25951. doi:10.3386/w25951.

[r21] African Development Bank Group. Tracking Africa’s Progress in Figures. 2014. https://www.afdb.org/fileadmin/uploads/afdb/Documents/Publications/Tracking_Africa%E2%80%99s_Progress_in_Figures.pdf.

[r22] McEvoy CS, Ross‑Li D, Norris EA, Ricca RL, Gow KW. From far and wide: Geographic distance to pediatric surgical care across Canada. J Pediatr Surg. 2020;55(5):908–912. doi:10.1016/j.jpedsurg.2020.01.036. 32063366

[r23] Global Health Policy. Breaking Down the U.S. Global Health Budget by Program Area. 2023. Accessed May 5, 2024. https://www.kff.org/global-health-policy/fact-sheet/breaking-down-the-u-s-global-health-budget-by-program-area/.

[r24] National Academies of Sciences, Engineering, and Medicine. Global Health and the Future Role of the United States. Washington, DC: The National Academies Press; 2017:24737. doi:10.17226/24737. 29001490

[r25] Vervoort D, Genetu A, Kpodonu J. Policy prioritisation to address the global burden of rheumatic heart disease. Lancet Glob Health. 2021;9(9):e1212. doi:10.1016/S2214-109X(21)00352-1. 34416209

[r26] Dieleman JL, Graves CM, Templin T, et al. Global health development assistance remained steady in 2013 but did not align with recipients’ disease burden. Health Aff. 2014;33(5):878–886. doi:10.1377/hlthaff.2013.1432. 24714869

[r27] Dieleman JL, Yamey G, Johnson EK, Graves CM, Haakenstad A, Meara JG. Tracking global expenditures on surgery: Gaps in knowledge hinder progress. Lancet Glob Health. 2015;3:S2–S4. doi:10.1016/S2214-109X(15)70075-6. 25926316

[r28] Meara JG, Leather AJM, Hagander L, et al. Global Surgery 2030: Evidence and solutions for achieving health, welfare, and economic development. Lancet. 2015;386(9993):569–624. doi:10.1016/S0140-6736(15)60160-X. 25924834

[r29] Roth GA, Johnson C, Abajobir A, et al. Global, regional, and national burden of cardiovascular diseases for 10 causes, 1990 to 2015. J Am Coll Cardiol. 2017;70(1):1–25. doi:10.1016/j.jacc.2017.04.052. 28527533 PMC5491406

[r30] Global Burden of Disease Collaborative Network. Global Burden of Disease Study 2019 (GBD 2019) Healthcare Access and Quality Index 1990–2019. Published online 2022. doi:10.6069/97EM-P280.

[r31] Nguyen N, Jacobs JP, Dearani JA, et al. Survey of nongovernmental organizations providing pediatric cardiovascular care in low‑ and middle‑income countries. World J Pediatr Congenit Heart Surg. 2014;5(2):248–255. doi:10.1177/2150135113514458. 24668973 PMC4276142

[r32] Vervoort D, Guetter CR, Munyaneza F, et al. Non‑governmental organizations delivering global cardiac surgical care: A quantitative impact assessment. Semin Thorac Cardiovasc Surg. 2022;34(4):1160–1165. doi:10.1053/j.semtcvs.2021.08.010. 34407434

[r33] Ahmed F, Grade M, Malm C, Michelen S, Ahmed N. Surgical volunteerism or voluntourism – Are we doing more harm than good? Int J Surg. 2017;42:69–71. doi:10.1016/j.ijsu.2017.04.020. 28433757

[r34] Molloy FJ, Nguyen N, Mize M, et al. Medical missions for the provision of paediatric cardiac surgery in low‑ and middle‑income countries. Cardiol Young. 2017:27(S6):S47–S54. doi:10.1017/S104795111700261X. 29198262

[r35] Dearani JA, Jacobs JP, Bolman RM, et al. Humanitarian outreach in cardiothoracic surgery: From setup to sustainability. Ann Thorac Surg. 2016;102(3):1004–1011. doi:10.1016/j.athoracsur.2016.03.062. 27319988

[r36] Dearani JA, Neirotti R, Kohnke EJ, et al. Improving pediatric cardiac surgical care in developing countries: Matching resources to needs. Semin Thorac Cardiovasc Surg Pediatr Card Surg Annu. 2010;13(1):35–43. doi:10.1053/j.pcsu.2010.02.001. 20307859

[r37] Shalabi HT, Price MD, Shalabi ST, et al. Mobile gastrointestinal and endoscopic surgery in rural Ecuador: 20 years’ experience of Cinterandes. Surg Endosc. 2017;31(12):4964–4972. doi:10.1007/s00464-016-4992-9. 28639040

[r38] Sangameswaran R, Verma G, Raghavan N, Joseph J, Sivaprakasam M. Cataract surgery in mobile eye surgical unit: Safe and viable alternative. Indian J Ophthalmol. 2016;64(11):835. doi:10.4103/0301-4738.195599. 27958207 PMC5200986

[r39] La Chaine De L’espoir. 2021 Annual Activity Report. Published online 2021. Accessed June 10, 2024. https://www.chainedelespoir.org/sites/default/files/ra_uk_2021.pdf.

