# Supplementary material for: Estimating Congenital Cardiac Surgical Need in Africa Using Geographic Distribution of Surgeons
Source: Ann Glob Health. 2025 Jun 25;91(1):36. doi: 10.5334/aogh.4692 (PMC12227093; doi:10.5334/aogh.4692)
Supplement: Supplementary Figure 1. — ICCR for each of the 63 Congenital Cardiac Surgical Catchment Areas Across Africa Operating at 75% U.S. Case Capacity. [file agh-91-1-4692-s1.pdf]

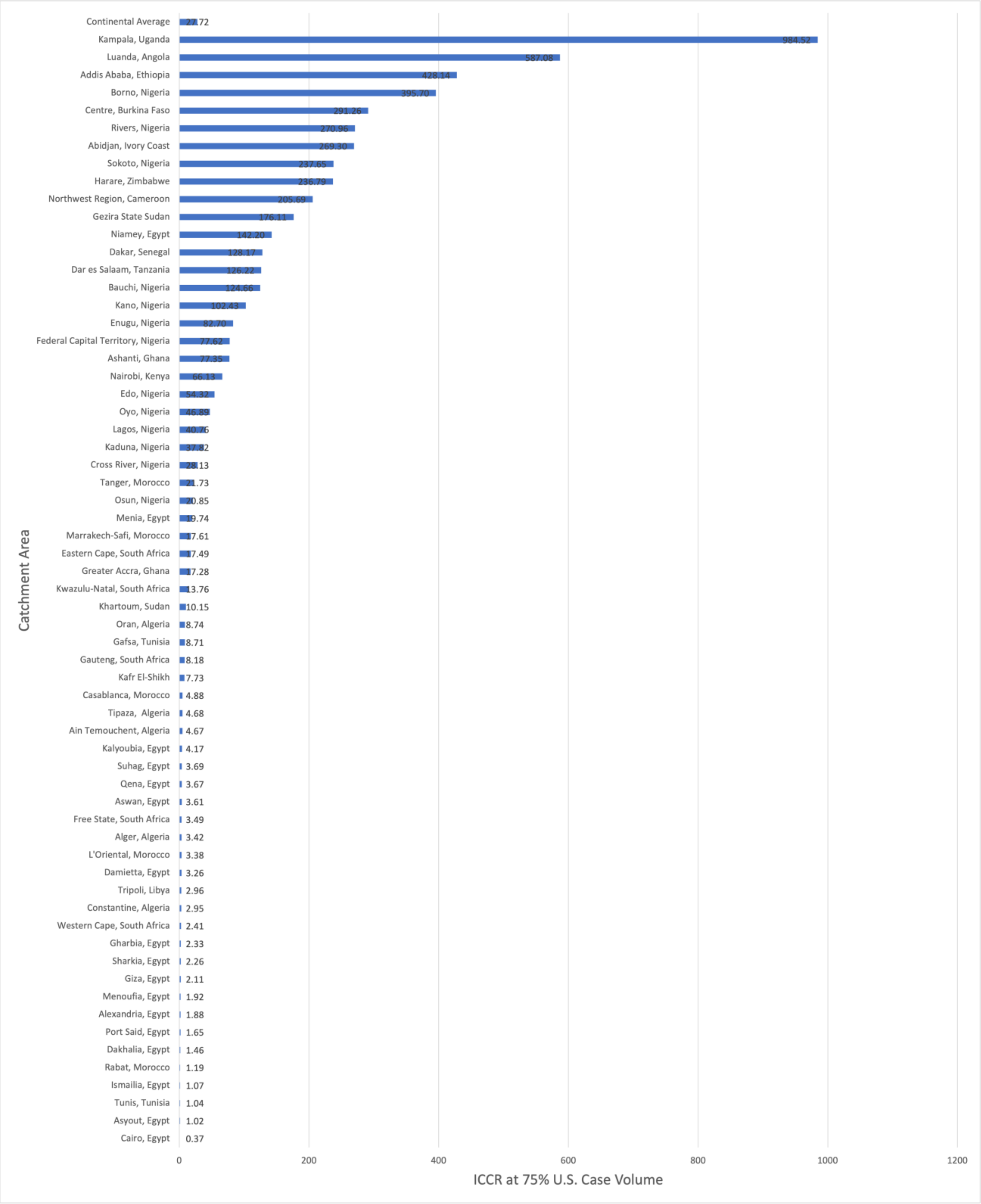

Supplementary Figure 1: ICCR for Each of the 63 Congenital Cardiac Surgical Catchment Areas Across Africa operating at 75% U.S. Case Capcity. *ICCR: Incident Case to Capacity Ratio*
