# Supplementary material for: Estimating Congenital Cardiac Surgical Need in Africa Using Geographic Distribution of Surgeons
Source: Ann Glob Health. 2025 Jun 25;91(1):36. doi: 10.5334/aogh.4692 (PMC12227093; doi:10.5334/aogh.4692)
Supplement: Supplementary Figure 3. — Tabulated Rates of CHD Incidence for Each Congenital Cardiac Surgical Catchment Area, CHD: Congenital Heart Disease. [file agh-91-1-4692-s3.pdf]

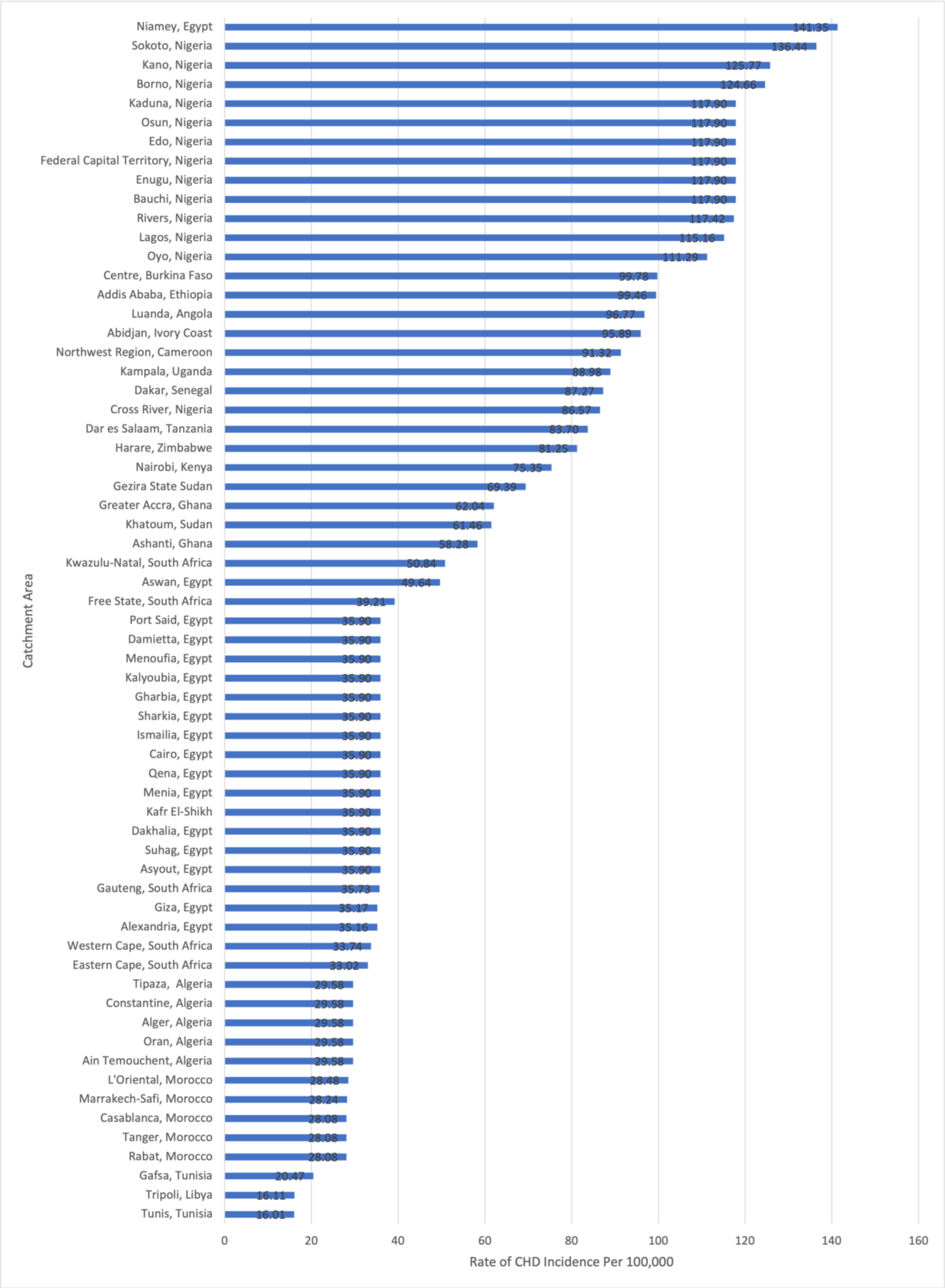

Supplementary Figure 3: Tabulated Rates of CHD Incidence for Each Congenital Cardiac Surgical Catchment Area, *CHD: Congenital Heart Disease*
